# How does dental implant macrogeometry affect primary implant stability? A narrative review

**DOI:** 10.1186/s40729-023-00485-z

**Published:** 2023-07-05

**Authors:** Diana Heimes, Philipp Becker, Andreas Pabst, Ralf Smeets, Annika Kraus, Amely Hartmann, Keyvan Sagheb, Peer W. Kämmerer

**Affiliations:** 1 Department of Oral and Maxillofacial Surgery, University Medical Center Mainz, Augustusplatz 2, 55131 Mainz, Germany; 2 Department of Oral and Maxillofacial Surgery, Federal Armed Forces Hospital, Rübenacherstraße 170, 56072 Koblenz, Germany; 3 Department of Oral and Maxillofacial Surgery, University Medical Center Hamburg-Eppendorf, Martinistraße 52, 20246 Hamburg, Germany; 4 Department of Oral and Maxillofacial Surgery, Division of “Regenerative Orofacial Medicine”, University Medical Center Hamburg-Eppendorf, Hamburg, Germany; 5 Department of Prosthetic Dentistry, University Medical Center Mainz, Augustusplatz 2, 55131 Mainz, Germany; 6 Private Practice for Oral Surgery, Echterdinger Straße 7, 70794 Filderstadt, Germany

**Keywords:** Implant geometry, Primary stability, Macro-design, Thread design, Implant length, Implant diameter, Narrative review

## Abstract

**Purpose:**

The macrogeometry of a dental implant plays a decisive role in its primary stability. A larger diameter, a conical shape, and a roughened surface increase the contact area of the implant with the surrounding bone and thus improve primary stability. This is considered the basis for successful implant osseointegration that different factors, such as implant design, can influence. This narrative review aims to critically review macro-geometric features affecting the primary stability of dental implants.

**Methods:**

For this review, a comprehensive literature search and review of relevant studies was conducted based on formulating a research question, searching the literature using keywords and electronic databases such as PubMed, Embase, and Cochrane Library to search for relevant studies. These studies were screened and selected, the study quality was assessed, data were extracted, the results were summarized, and conclusions were drawn.

**Results:**

The macrogeometry of a dental implant includes its surface characteristics, size, and shape, all of which play a critical role in its primary stability. At the time of placement, the initial stability of an implant is determined by its contact area with the surrounding bone. Larger diameter and a conical shape of an implant result in a larger contact area and better primary stability. But the linear relationship between implant length and primary stability ends at 12 mm.

**Conclusions:**

Several factors must be considered when choosing the ideal implant geometry, including local factors such as the condition of the bone and soft tissues at the implant site and systemic and patient-specific factors such as osteoporosis, diabetes, or autoimmune diseases. These factors can affect the success of the implant procedure and the long-term stability of an implant. By considering these factors, the surgeon can ensure the greatest possible therapeutic success and minimize the risk of implant failure.

**Graphical Abstract:**

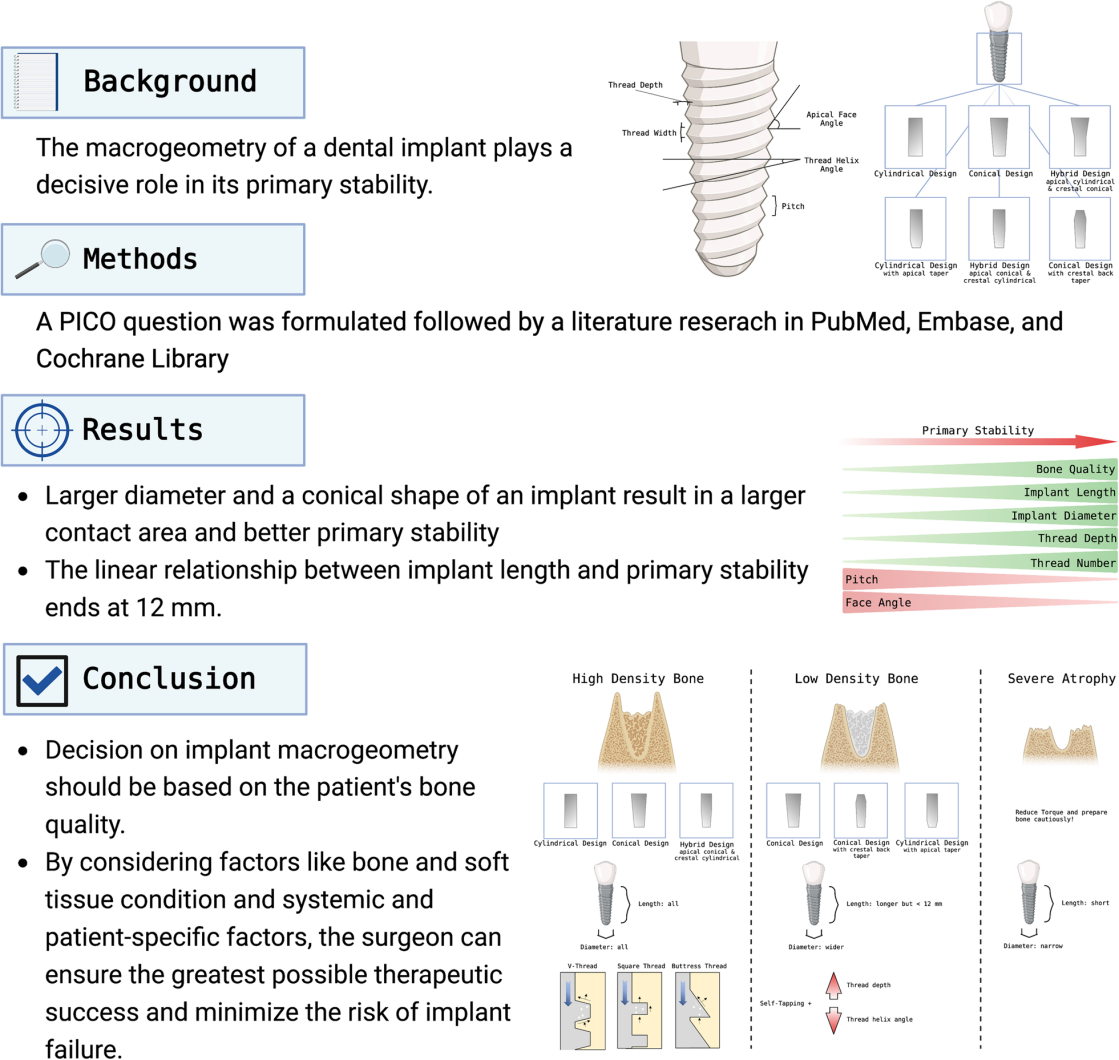

## Background

Implantology is a branch of dentistry that deals with dental implants to replace missing teeth. A dental implant is a screw-shaped post made of a biocompatible material, such as titanium surgically inserted into the jawbone to support a replacement tooth or bridge [1, 2]. Dental implants are a popular and effective treatment option for patients who have lost teeth due to trauma, decay, or other causes. They offer many advantages over tooth replacement options such as dentures or bridges, which include improved function, durability, and esthetics. Dental implants can last for decades or even a lifetime [3–5] with proper care and maintenance. One of the most important advantages of dental implants is the high survival and success rates. Studies have shown that dental implants have a success rate of over 95%, meaning they are successful in most cases. [3]. This high success rate is due to the biocompatibility of the implant material, which allows it to fuse with the jawbone and form a stable foundation for restoration. When a dental implant is placed it stimulates the surrounding bone tissue, helps maintain bone density, and prevents bone loss [4].

Dental implant placement can be categorized into immediate, delayed, and late. Each category refers to the time frame between the extraction of a tooth and the placement of a dental implant [5]. Immediate placement of dental implants involves placing an implant immediately after tooth extraction. This procedure is usually performed when the extraction site has sufficient bone volume and the implant can be stabilized. The established and well-documented benefits of immediate implants, like reduced treatment time, high patient satisfaction, comfort, and high survival rates, implicate that it is a reliable treatment method [1, 3, 6]. Of course, the position of the immediate implant is crucial. Bone formation and bone preservation of the buccal wall were shown to be influenced by the initial labio-palatal socket dimension [7]. New protocols like task-autonomous robotic systems in immediate implant placement seem to be beneficial [8]. But studies have shown no evidence for any effect of the implant’s macrogeometry on the accuracy of guided implant insertion [9]. With growing scientific evidence and experience in the field, according to the authors, the indications for immediate implant placement will increase in the future.

Delayed implant placement is performed several weeks to months after tooth extraction. This procedure is usually performed when the extraction site needs healing time to ensure the bone is dense enough to support the implant. Delayed implant placement allows adequate healing time and reduces the risk of implant failure [10]. Late implant placement refers to the placement of an implant after a long period has passed since tooth loss. This can be due to various factors, including patient preferences, systemic or local diseases, or other factors that may have prevented earlier implant placement. Late implant placement may require additional augmentation procedures such as bone grafting or sinus lift to ensure adequate bone volume for the implant [11]. The choice of implant placement type depends on several factors including the indication for tooth replacement, the quality and quantity of remaining bone, and the patient's overall health. Implant design is critical in achieving the primary and secondary stability essential for successful osseointegration and long-term implant success. Several important design factors can affect implant stability including implant diameter, implant length, implant thread design, implant surface roughness, and implant shape [12–15]. This narrative review aimed to find an adequate answer to the question: What influence does the macro-design have on the primary stability of dental implants?

## Methods

The first step was to define the research question or goal of the review clearly. For example, “What is the impact of the macrogeometry of the implant on primary stability?”. Electronic databases, such as PubMed, Embase, and the Cochrane Library, were then used to search for relevant studies. Search terms also included "dental implant", "macrogeometry", "primary stability", "implant shape", "implant size" and "implant surface properties". Reference lists of retrieved articles were reviewed to find other relevant studies. A list of relevant studies was created, titles and abstracts were screened for inclusion criteria, and the methodological quality of the included studies was assessed using appropriate tools such as the Cochrane Risk of Bias Tool or the Newcastle–Ottawa Scale for observational studies. The next step was to extract relevant data from the included studies in a standardized form. This data included information on study design, sample size, intervention, outcome measures, and outcomes. After analyzing the extracted data, patterns and trends in the results were identified. Finally, based on the summarized results, conclusions were drawn on the influence of the implant macrogeometry on the primary stability.

## Results and discussion

### Literature review on the effect of dental implant macrogeometry on primary implant stability

As a limitation, the terms "short", "long", "standard”, "narrow", "wide”, and "regular” dental implants are not defined consistently [16]. Several studies have investigated the effects of dental implant macrogeometry on primary implant stability. A literature review of these studies suggests that implant macrogeometry may be essential in achieving primary implant stability. One study compared the primary stability of dental implants with different macrogeometries, including cylindrical, conical, and hybrid designs. The study found that the hybrid design had the greatest primary stability, followed by the conical design and then the cylindrical design [17]. Another study compared the primary stability of dental implants with different thread designs including V-shaped and square threads. The study found that implants with V-shaped threads had higher primary stability than those with square threads [18]. The literature suggests that implant macrogeometry can significantly influence primary implant stability. Hybrid and conical designs provide the greatest primary stability, while thread design may also play a role. However, further studies are needed to investigate the effect of macrogeometry on primary implant stability and to determine the optimal macrogeometry design for different clinical situations.

#### What is the impact of implant body shape on primary stability?

**Fig. 1:**
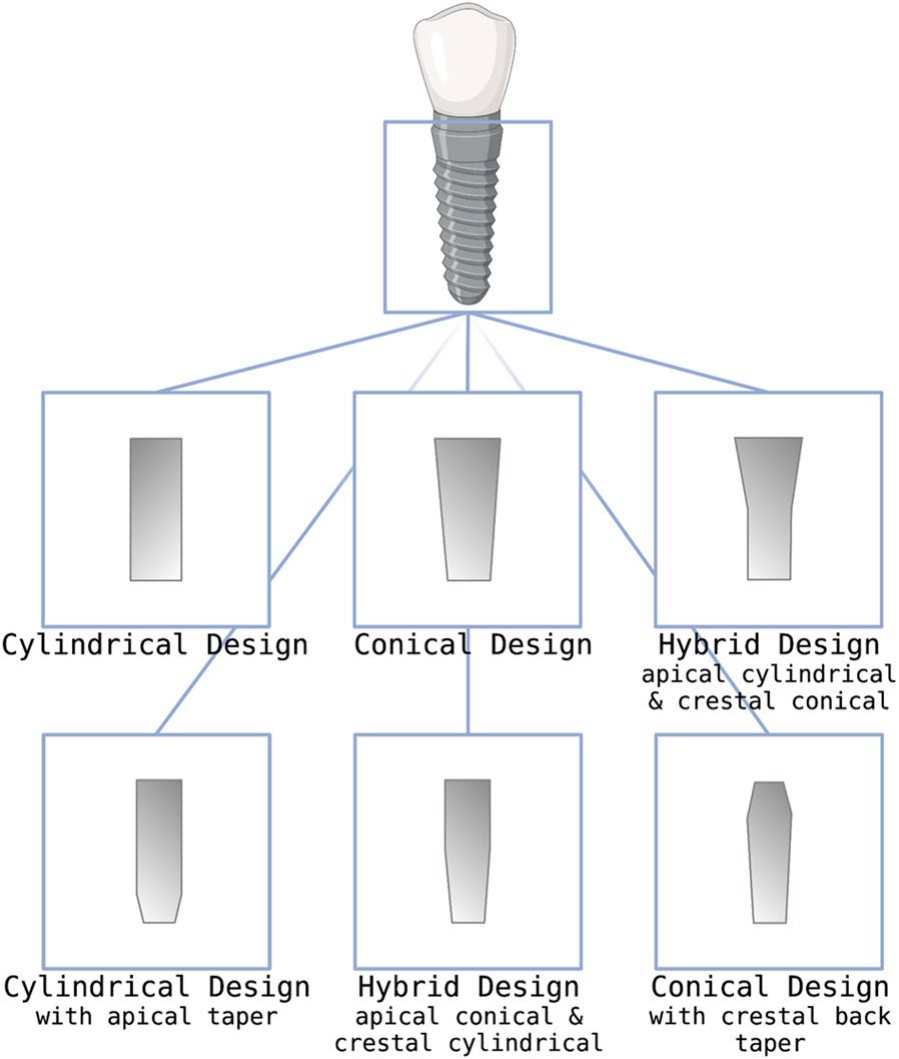
Different implant designs commercially available. Created with BioRender.com

An essential distinction is made between cylindrical and conical implants; there are now numerous mixed forms (see Fig. 1) [19]. Tapered implants are primarily anchored by lateral and vertical bone compression, while cylindrical implants transmit static friction to the implant base along the implant axis [20]. Tapered implants are becoming increasingly popular given the ease of clinical use, reduced drilling sequences, potentially shorter healing times, and decreased surgical trauma [21]. The interplay of loading magnitude, loading direction, frequency, bone quality, and quantity, and the ability of cells to respond to loading affect the bone's response. Tapered and threaded implants distribute the load better than cylindrical implants [22]. In addition, buccal/facial bone perforation is less likely with conical-shaped implants due to their anatomical design [21, 23]. In a human ex vivo study, O-Sullivan et al. [23] demonstrated that conical implants have a significantly higher primary stability than the standard Brånemark design. These results were also confirmed by Merideth et al. in a clinical setting [3, 24]. Tapered implants exert lateral compressive forces on the cortical bone, which may be a significant reason for their increased primary stability [12, 25]. In addition to the high primary stability, conical implants are thought to have improved osseointegration properties [12, 26, 27]. Apically conical implants show increased primary stability among the hybrid forms due to more substantial crestal compression. In contrast, crestal parallel implants or back taper designs better relieve the bone [19]. Self-tapping threads can also contribute to increasing primary stability.

Immediate implant placement requires high primary stability, so conical implant systems with double threads and a low thread helix angle should be selected [19]. The influence of the shape of the implant body on primary stability is an essential aspect of implantology [28]. As mentioned earlier, tapered or conical implants provide greater primary stability than cylindrical implants because they can distribute forces more evenly and incorporate more bone [29]**.** However, there are some challenges associated with the use of tapered or conical implants. One challenge is that these implants may require a more invasive surgical approach to prepare the implant site and to ensure proper placement [30]. According to the Group 1 ITI Consensus Report: The influence of implant length and design and medications on clinical and patient-reported outcomes of 2018, evidence shows that both tapered and non-tapered implants demonstrate satisfactory performance with respect to marginal bone levels at 3 years. Based on the consensus statements, tapered and non-tapered implants can be used according to the operator's preference. Tapered implants might be beneficial in clinical situations to avoid injuring anatomical structures or causing apical fenestrations. Furthermore, in situations where increased insertion torque is needed, tapered implants may be considered; however, the clinical significance of the implant shape on long-term success is still unclear [31].

#### How does implant length affect primary stability?

In addition to the implant shape, the macro-design is determined by the length and thickness of the implant. Long and thick dental implants are intended for use with significant bone loss or to support a more extensive prosthetic restoration. These implants are typically used in areas where conventional implants are not suitable due to insufficient bone volume or density. Long implants are usually defined as those 15 mm or more in length. These implants offer a larger surface area for osseointegration and can attach to more bone, resulting in greater primary stability. Long implants are often used in the posterior maxilla or mandible where bone density and height are typically lower [21].

Thick implants, on the other hand, are designed for greater mechanical stability and can withstand higher loads. These implants are larger in diameter and can be used where larger prosthetic restorations need to be supported or where there is high occlusal loading. Thick implants are often used in the posterior maxilla or mandible, where there is usually greater bone density and height. A potential challenge with using long and wide implants is that they require a more invasive surgical approach and may be more difficult to place accurately due to their size. Bruggenkate et al. reported a 10-year survival rate of 94% based on a total of 253 short (6 mm) implants placed. They recommended the placement of short and long implants in combination, especially when implant restoration in less dense bone is planned [1]. Barikanie et al. concluded from an in vitro study that primary stability increased significantly with implant length. However, it should be noted that implant lengths between 10 and 16 mm were examined in this study [32]. In contrast, Staedt et al. showed that different implant lengths and diameters do not achieve significantly different primary stability parameters in dense bone [33]. Dense bone refers to bone tissue with a higher density and mineral content than other types of bone tissue. This type of bone is stronger and more resistant to fractures and different types of damage [34]. This article adopts the ITI Consensus definition of a short implant of ≤ 6 mm. The ITI Consensus Report on the influence of implant length on clinical and patient-reported outcomes concluded that short implants (≤ 6 mm) exhibit similar survival rates compared to longer implants after 1 to 5 years. Based on ten randomized-controlled studies, a mean survival rate of 96% for short compared to 98% for longer implants over 1 to 5 years was calculated. Furthermore, prosthodontic restorations' survival was comparable in both groups after 1 to 5 years of function. A meta-analysis showed that, after a 1-, 5- and 10-year follow-up, short implants (≤ 6 mm) and longer implants (≥ 8.5 mm) showed no significant differences in survival rates even in the non-atrophic jaw without the need for bone augmentation. However, the data regarding implant geometry (length and diameter) are very heterogeneous [31, 35]. The ITI Consensus Group recommends using short implants where bone grafting procedures are contraindicated/where the morbidity of such procedures should be avoided or to reduce treatment time. Furthermore, they may be indicated where the possibility of damaging adjacent structures, like the maxillary sinus, nerves, or other implants, can be reduced. Implants longer than 6 mm should be preferred when placed without increased surgical risk [31]. In this context, not only the length of the implant itself should be considered. The ratio of crown length to implant length should also not be ignored. The stress on the peri-implant must be considered, as this can increase as the crown-to-implant ratio increases, especially in the case of immediate loading concepts [36–38]. Three-dimensional finite-element analyses indicate beneficial stress values in tissue-level compared to bone-level designs [39].

#### How does implant diameter affect primary stability?

Narrow-diameter implants are defined as dental implants with a diameter of ≤ 3.5 mm. They can be further divided into category 1, narrow-diameter implants with a diameter < 2.5 mm (mini-implants; mostly one-piece implants), category 2, with a diameter of 2.5 mm to < 3.3 mm and category 3, with a diameter of 3.3 mm to 3.5 mm [40]. Implants with a diameter ≥ 5 mm are referred to as wide-diameter implants [40]. Animal studies suggest a larger diameter is associated with greater primary stability [41–43]. Since stress is applied to the implant shoulder, the implant diameter is considered the most critical parameter for stress and load distribution [44, 45]. Increasing the implant diameter increases both primary stability, and functional surface area, contributing to better load distribution. However, a considerable number of studies have shown that implants with reduced diameters can also develop sufficient primary stability in reduced-quality bone. Rossa et al. reported similar results in their retrospective evaluation of failure rates in dental implants [46]. Accordingly, an increased probability of early dental implant failure has been observed with implants in the mandible—especially in the posterior part of the jaw. Contrary to this, a higher patient age, a localization within the maxilla, and a greater implant length was associated with late dental implant failure. Javed et al. assumed implant diameter to play a secondary role in implant survival and suspected the surface quality to be much more relevant [11]. Among these are retention sites or micro-threads at the implant shoulder, which have led to better load distribution in the alveolar ridge [47]. Furthermore, Kämmerer et al. showed that mini-implants could also achieve satisfactory results. The strict reduction of insertion torque and the best possible preparation of the bone were described as particularly relevant [48]. Some studies attribute a lower survival rate to wide-diameter implants. In a meta-analysis, Lee et al. confirm a promising 5-year survival rate for wide-diameter implants. But to make a statement with strong evidence on this question, further high-quality studies are needed [41]. The data regarding the effect of the implant’s diameter on the survival and success rate are heterogenous [31, 49]. But the ITI Consensus Conference in 2018 reported similar survival rates of narrow implants with a diameter of 2.5 mm and larger than standard diameter implants [31]. Since the stress is concentrated around the implant neck, where bone loss occurs at an early stage, it is now assumed that diameter becomes a more decisive factor as soon as implant length is sufficient. Particularly in the posterior region, two complementary, unfavorable conditions appear: on the one hand, masticatory forces are more than 300% higher than in other tooth regions, and on the other hand, the posterior region often presents a comparably low bone quality in the maxilla. Considering this, conventional protocols based on increasing the surface area merely by changing the implant diameter are insufficient. While such a concept can only contribute to a 30% increase in surface area, up to a 300-fold increase in surface area is possible by modifying the diameter and thread type [1].

#### How does the thread design affect primary stability?

**Fig. 2:**
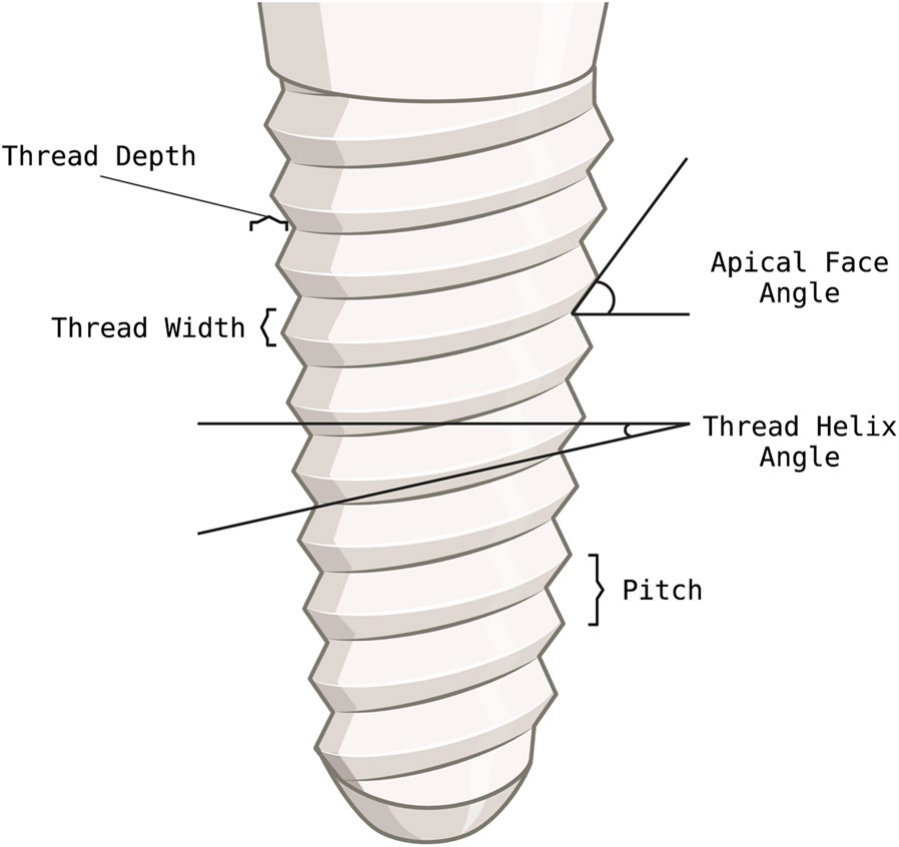
Characteristics of the implant macro design. Thread helix angle: angle between the thread helix and the horizontal to the longitudinal axis of the implant. Apical face angle: angle between the thread face and the horizontal to the longitudinal axis of the implant. Pitch: distance between the center of a thread to the next thread in the longitudinal axis of the implant or implant length divided by the number of threads. Thread depth: distance between the outer contour of the thread and the implant base body. Thread width: distance between the most coronal and the most apical portion of the same thread (Mod. according to [60]). Created with BioRender.com

Threads increase bone–implant contact area, primary stability, implant surface area, and better load distribution [50]. Here, the thread design is a decisive factor for the initial mechanical primary stability and the subsequent biological secondary stability of the implant [51–53]. Thread depth, width, pitch, face angle, and helix angle variations are possible (see Fig. 2). Thread shapes include V-shaped, square, buttress, and helical designs. The insertion of fewer threaded implants was reported to be smoother, which could be an advantage in denser bone [54].

Dental implants with a small pitch automatically have more threads per implant length and thus a greater implant surface, which could result in a better load distribution [55–57]. These macro-design parameters are interdependent, and all increase primary stability. A current systematic review states that the study situation on thread design is very heterogeneous. In summary, however, it was found that higher bone–implant contact was found with the mere presence of threads, with implants with a smaller pitch, with V-threads with implants with smaller thread pitches (0.6 to 0.8 mm on average), and with a larger thread depth [12]. Especially aggressive self-tapping threads have been reported to increase primary stability [58, 59].

#### Thread depth

Threaded implants were initially developed to allow greater compression of the cortical bone in sites of poor bone quality [61]. The thread depth is defined by the ratio of the outer contour to the main body of the implant. It indicates the distance by which the coils protrude from the main body of the implant. The longer this distance, the more the surface and the load distribution increase [20, 47]. Greater thread depths could be advantageous due to the increased functional surface, especially with softer bone and high occlusal forces, and increase primary stability in these situations. Still, greater thread depths may also reduce insertion accuracy [62–64]. The implant design also comes up against biological limits here since, with very deep threads, it is impossible to guarantee adequate vascular supply to the bone that extends to the thread's root. With a considerable thread depth, it is, therefore, advisable to pre-tap threads to avoid excessive compressive stress on the surrounding bone [20, 47]. The more threads an implant has and the deeper they are, the greater the functional surface of the implant [1, 65]. Studies have shown implants with a progressive thread to have a higher bone–implant contact area histomorphological and radiologically compared to cylindrical designs and to provide a higher primary stability [66].

#### Thread helix angle

The thread helix angle is defined as the angle between the thread grind and the plane perpendicular to the longitudinal axis of the implant [60]. It defines the propulsion of the implant when it is inserted. The higher the thread helix angle, the fewer rotations the implant requires to insert it to its entire length. A a high thread helix angle can also lead to longitudinal rotation of the implant under axial loading [20]. Finite element analysis has shown that faster placement of multi-threaded implants is associated with a higher implant failure rate [60]. There are systems without a thread helix angle and multiple threads available, which differ primarily in the insertion technique [20]. In multi-threaded implants, more than one thread runs parallel to another thread. This configuration results in faster implant placement. For example, double-threaded implants cover twice as much insertion distance per rotation as single-threaded implants, with parameters of the macro-design being otherwise equal. However, this seems to come at the expense of primary stability [60, 67].

#### Thread width, thread shape and face angle

**Fig. 3:**
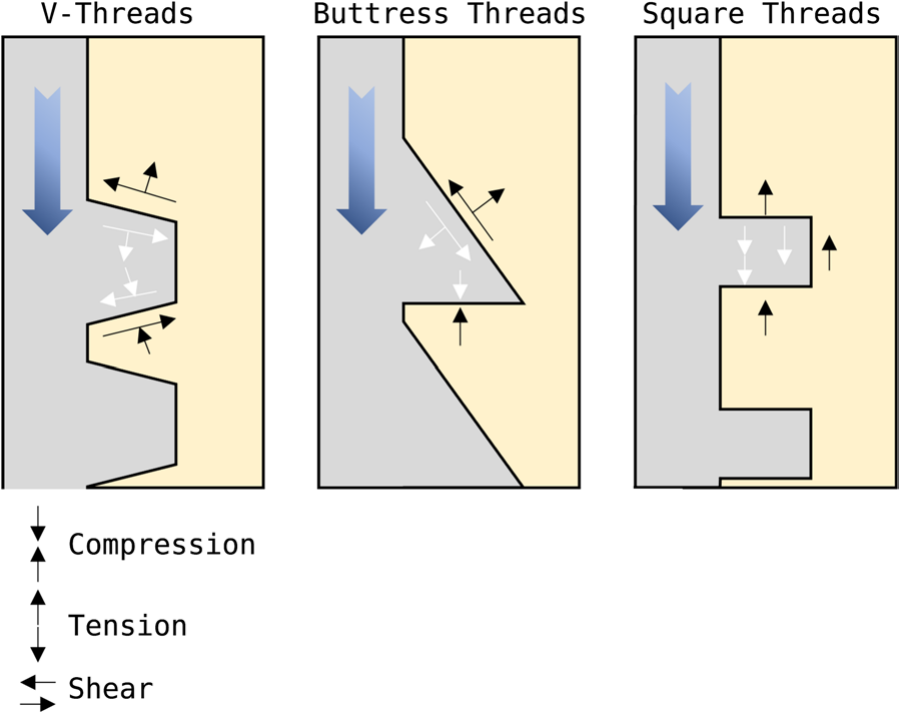
Forces generated by axial loading of the implant at the bone–implant contact surface (Mod. according to [60]). Created with BioRender.com

The thread width determines how the implant is guided when inserted and largely depends on the thread's shape (see Fig. 2). V-shaped and wide square implants can cause significantly less stress on the cancellous bone than thin square shapes. In cortical bone, no difference could be detected. Under dynamic loading, bone density is highest directly under the whorl. This confirms the implicit correlation between compressive load and bone density [60]. With square and buttress threads, axial forces are mainly distributed as compressive forces. On the other hand, with V-shaped reverse buttress threads, axial forces will be converted into shear and compressive forces. Using an ample thread width often requires thread pre-cutting of the bone cavity and also ensures easy implant guidance. The advantage of thread cutting is a significant reduction in insertion torque. On the other hand, self-tapping implants often lead to increased primary stability, especially in softer bone or fresh extraction sockets [20, 51]. Multiple cutting threads could also provide higher primary stability in bone with low density [68]. In addition, square threads seem advantageous for the implant's immediate loading [69]. A thread width of 0.18–0.3 mm and a thread depth of 0.34–0.5 mm were proven advantageous. The thread depth is more susceptible to stress maxima than the thread width [60]. The face angle is the angle between the thread face and the horizontal to the longitudinal axis of the implant. Each thread has an apical and coronal surface. Thread face angle is also directly related to thread form, with V-shaped threads having a face angle of 30 degrees, while reverse buttress threads have a face angle of just 15 degrees. This is why implants with a V-shaped thread develop significantly more shear forces than implants with a smaller face angle, which predisposes to defect formation [60]. This thread face angle directly determines the direction of loading from the implant towards the surrounding bone [70] (see Fig. 3). A more than 0.8 mm pitch is considered ideal, and greater thread-to-thread spacing is associated with greater resistance to vertical loads [60].

#### Additional threads

The use of smooth implant shoulders was originally used to reduce plaque accumulation when the crestal portion of the implant was placed above bone level (tissue-level implants). However, the problem with a smooth implant shoulder is that putting it below bone level under shear stress can result in marginal bone loss with pocketing. For this reason, implants with retentive elements and threads around the implant shoulder were developed, leading to better integration in the cortical bone and, thus, a reduction in bone resorption [71]. In finite-element analyses, this theory was confirmed by Abuhussein et al. [59], whereas ex vivo and in vivo studies show ambiguous data. It is still uncertain whether crestal-located threads contribute better load distribution and thus to the desired preservation of crestal bone or its degradation [3]. In a systematic review, Lovatto et al. found that such micro-threads protect hard and soft tissue [72]. However, a recent prospective, randomized, clinically controlled, multicenter study found no differences between machined tissue-level and roughened neck bone-level implants regarding peri-implant bone loss, peri-implantitis rate, implant survival rate, and hard and soft tissue situation [73]. The original 1965 Branemark implant had a V-shaped thread designed for better placement within the pre-drilled osteotomy cavity. Threads have come a long way since then. Implants are currently being produced with double or triple threads, which slide more quickly into the osteotomy cavity and are intended to offer increased initial primary stability. Although their advantage of faster insertion, double-threaded implants with a higher lead angle may also cause bone tissue damage because they need to be inserted with increased torque [74]. Therefore, they are particularly indicated in very soft bone. In addition, an extension of the thread area up to the implant tip can lead to an increase in primary stability [75]. So far, despite many studies on the different properties of the various thread types, there are no meaningful comparative studies [1].

## Conclusion

**Fig. 4:**
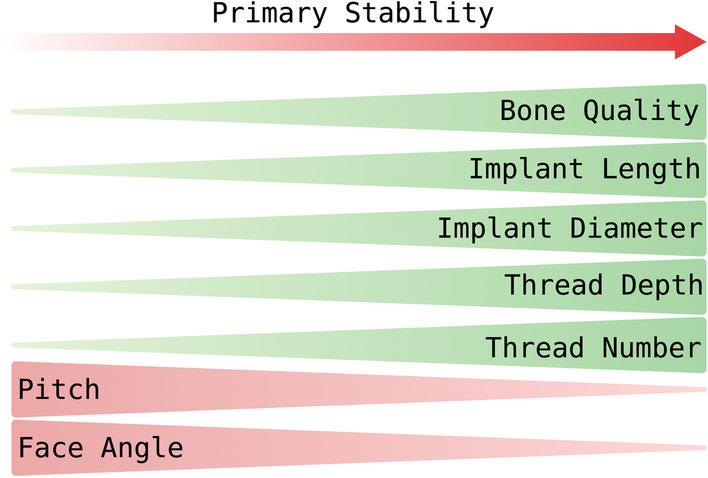
Factors influencing primary implant stability. See the text for an explanation. [11, 46, 60]. Created with BioRender.com

The shapes associated with high primary stability are conical, apical conical, and hybrid implants. With increasing length, the contact surface to the surrounding bone and, thus, the primary stability rises linearly. This linear relationship between implant length and primary stability ends at 12 mm. Conical implants are suitable for immediate implant placement protocols and in the anterior region, as they show better load distribution and less bone perforation with thin buccal bone thickness. In the atrophic jaw, implants with a reduced length and diameter are an alternative to augmentation procedures. With strict torque control and ideal bone preparation, good results can be expected even under challenging situations. Combining short implants with longer implants is recommended for cases with low bone density and reduced alveolar ridge height due to similar survival rates to increase implants' success rates. Macrostructures associated with a high effect on the bone-to-implant contact area and, thus, on primary stability are diameter and thread. A high thread depth seems advantageous when inserting with expected high occlusal forces and low bone density. V-shaped and square threads can reduce stress on the bone, while square and crossed threads may reduce compression. In addition, self-tapping threads can be recommended to increase primary stability, especially in low-density bone (Fig. 4).

Limitations of the narrative review are the inconsistency regarding the technical terms and the great heterogeneity of the included studies. Many studies assessing the effect of implant geometry on primary stability are ex vivo and finite-element analyses or retrospective clinical studies. There is a lack of prospective clinical studies analyzing differences in implant macrogeometry regarding primary stability and long-term success; therefore, no conclusions can be drawn in this regard.

**Fig. 5:**
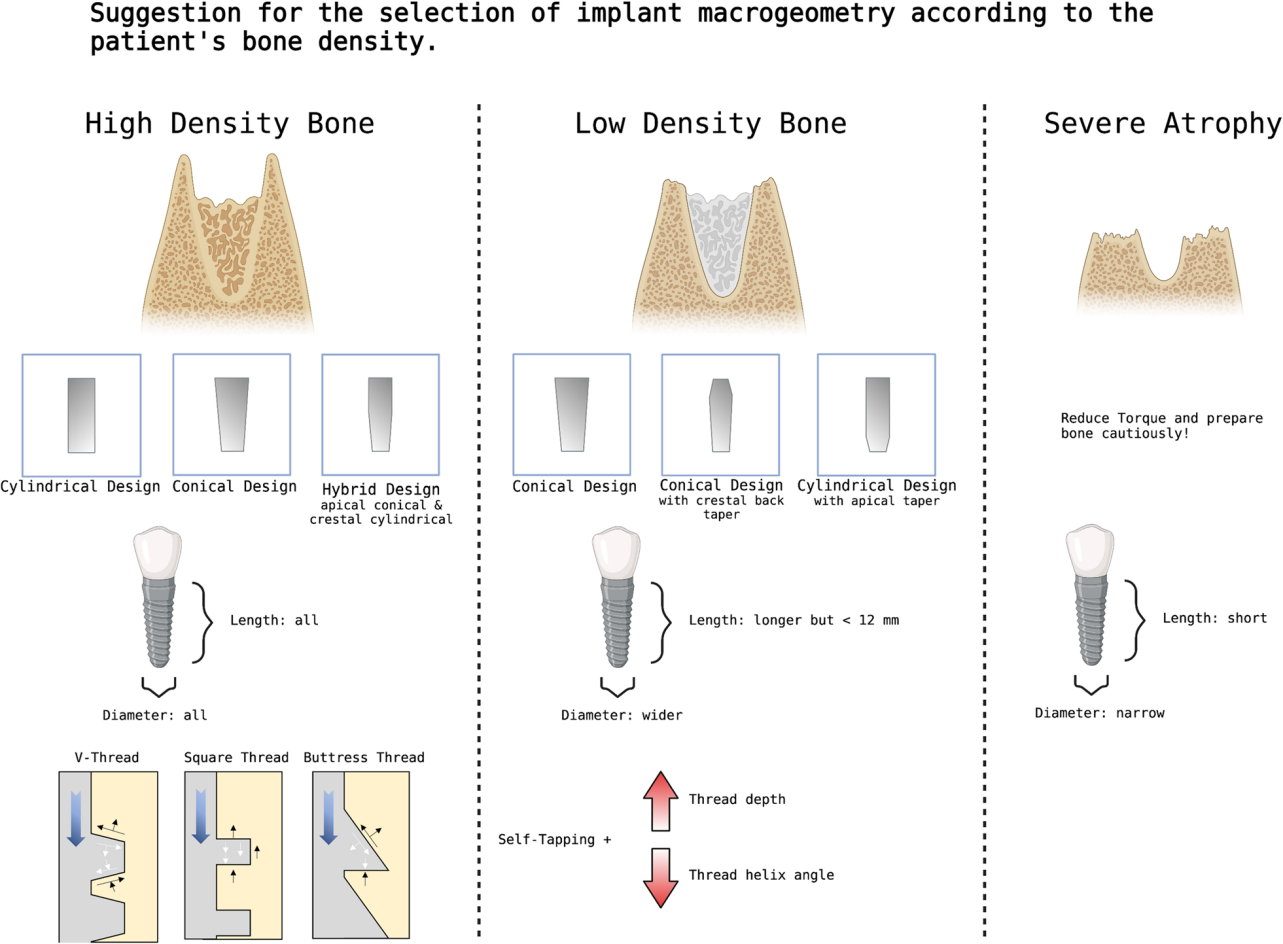
Suggestion for the selection of implant macrogeometry according to the patient's bone density. Created with BioRender.com

Figure 5 gives suggestions on the selection of the implant’s macrogeometry according to the patient's bone density. Overall, implants should be chosen individually for each case. With many different implant designs, it is not always easy to decide. The experience of the surgeon and how proficient he is with the respective implant design are also important. An implant design that is ideal for the individual case does not bring any benefit if the practitioner cannot deal with it in everyday life. Therefore, the patient's local and systemic factors, the surgeon's skill, and the technical possibilities must be considered. In addition, the evidence for many parameters that define the dental implant macrodesign is often still very limited, as there is currently a lack of high-quality studies.

## Data Availability

The datasets generated and analyzed during the current study are available online and upon reasonable request from the relevant author.
